# Transfusion Associated Peak in Hb HPLC Chromatogram – a Case Report

**DOI:** 10.4084/MJHID.2012.006

**Published:** 2012-01-21

**Authors:** Sonal Jain, Jasmita Dass, Hara Prasad Pati

**Affiliations:** Department of Hematology, All India Institute of Medical Sciences, New Delhi

## Abstract

High performance liquid chromatography (HPLC) and electrophoresis are commonly used to diagnose various hemoglobinopathies. However, insufficient information about the transfusion history can lead to unexpected and confusing results. We are reporting a case of Juvenile myelomonocytic leukemia (JMML) in which HbHPLC was done to quantify fetal hemoglobin (HbF). The chromatogram showed elevated HbF along with a peak in the HbD window. A transfusion acquired peak was suspected based on the unexpectedly low percentage of HbD and was subsequently confirmed using parental HbHPLC.

## Introduction

It is well known that incomplete history on test request forms sent to laboratories and inappropriate patient samples can lead to wrong diagnosis and hazardous consequences. Hemoglobin electrophoresis and High Performance Liquid Chromatography (HPLC) are routinely done to diagnose and classify hemoglobinopathies. Acquired and inherited conditions in which abnormal HPLC result can be seen include high fetal hemoglobin in Juvenile Myelomonocytic Leukemia (JMML), Diamond Blackfan Anemia (DBA) and Fanconi Anemia.^1^ Blood transfusion from donors with hemoglobinopathies which are clinically silent (e.g HbE, HbD, HbS) may lead to abnormal peaks or altered percentages of abnormal hemoglobins.

## Case

**Figure 1:**
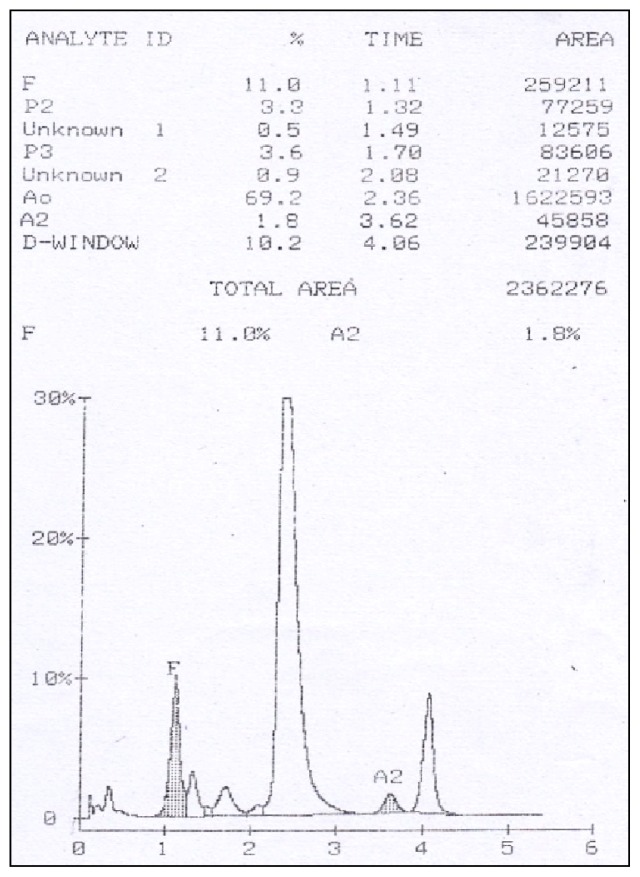
Hb HPLC of patient showing high HbF along with a peak in D window (RT – 4.06 minutes).

**Figure 2:**
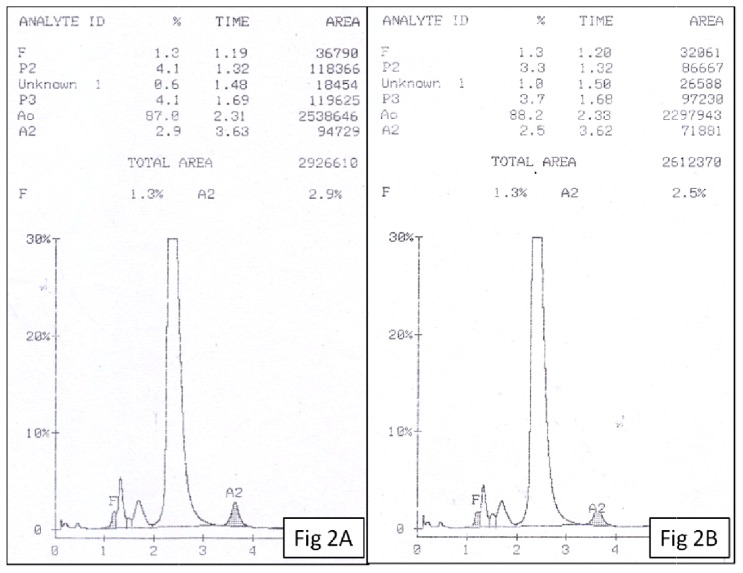
Hb HPLC of father (2A) and mother (2B) showing normal chromatogram.

A 7 year old female presented to the outpatient department with complaints of hepatosplenomegaly and lymphadenopathy for 3 months. Hemogram showed a hemoglobin of 9.5 gm/dl, total leukocyte count of 57.24 ×10^9^/L and platelet count of 16 ×10^9^/L. Peripheral smear showed 35% monocytes with an absolute monocyte count of 20 ×10^9^/L. Diagnosis of Juvenile myelomonocytic leukemia was suggested and Hb HPLC was advised to look for increased fetal haemoglobin. High Performance Liquid Chromatography (HPLC) was done using BioRad Variant II instrument with beta thalassemia short program. Hb HPLC chromatogram showed a raised HbF (11%) with HbA_0_ 69.2%, HbA_2_ 1.8% and a peak in D-window of 10.2% with a retention time of 4.06 minutes (Figure 1). The expected percentage of HbD in heterozygotes is ~40% but in our case it was significantly low. Hence, a possibility of transfusion acquired HbD was considered and Hb HPLC was done in both the parents. Both mother and father showed normal Hb HPLC (Figure 2A and 2B). The transfusion history was taken and it was discovered that the patient had received 3 units of PRBC one week prior to HPLC. Thus a final diagnosis of transfusion associated peak in D window was made.

There are only a few reports of abnormal hemoglobin peaks following blood transfusions.^2^–^5^ In these reports, the abnormal hemoglobins account for 0.8 – 14% of the total hemoglobin.^4^ The largest series by Kozarski et al reported 52 occurrences of transfusion associated peaks in 32 recipients. The possibility of transfusion associated peaks was suspected on the basis of low concentrations, transient nature or in one case, with presence of three different peaks.^4^

The incidental finding of such abnormal peaks may cause diagnostic and therapeutic dilemmas even to the most experienced, particularly so in patients with suspected hemoglobinopathies who have received multiple blood transfusions. To avoid this rare problem some authors have suggested screening of blood donors for hemoglobinopathies.^2^ In this case, we want to highlight that a high index of suspicion along with careful clinical evaluation, family history and required investigations in parents and siblings, transfusion history and evaluation of unexplainable Hb percentages are some key features which can be helpful in correctly diagnosing this rare possibility.
